# Parents' knowledge and awareness of physiological genu varum in Saudi Arabia

**DOI:** 10.25122/jml-2024-0404

**Published:** 2025-07

**Authors:** Omer Alrasheed, Amnah Alkhawajah, Rabab Alkhalaf, Ziyad Saad Alasmari, Mazen Mohammed Hakami, Mohammed Almarhabi

**Affiliations:** 1 Orthopedic Department, College of Medicine, King Faisal University, Kingdom of Saudi Arabia; 2 College of Medicine, King Faisal University, Kingdom of Saudi Arabia; 3 College of Medicine, King Khalid University, Abha, Saudi Arabia; 4 College of Medicine, Jazan University, Jazan, Saudi Arabia

**Keywords:** Genu varum, bowlegs, parental knowledge, awareness, musculoskeletal variations, epidemiology, Saudi Arabia

## Abstract

Genu varum is one of the most common lower limb musculoskeletal variations observed in Saudi Arabia. Understanding parents’ knowledge and perceptions of genu varum is essential for identifying gaps in awareness and improving early recognition and management through targeted educational interventions. This study aimed to assess the level of parental knowledge and awareness regarding physiological genu varum in Saudi Arabia. A questionnaire-based cross-sectional study was conducted among Saudi parents aged 18 years or older. Data were collected electronically via Google Forms, ensuring anonymity. The questionnaire, disseminated through various social media platforms, consisted of three sections: demographic information, general knowledge of genu varum, and awareness of physiological genu varum. A total of 1,222 respondents participated, with the majority being female (70.2%). Overall, 52.2% of parents demonstrated poor knowledge, while 47.8% had good knowledge of the condition. Parental knowledge was significantly associated with region, marital status, and having a child diagnosed with genu varum. In contrast, gender, age, income level, and nationality showed no significant influence. The current study revealed that approximately half of the surveyed parents demonstrated adequate knowledge about genu varum, particularly its physiological form and potential consequences. Higher knowledge was associated with being married and having a child with genu varum.

## INTRODUCTION

Genu varum (commonly known as bowlegs) is one of the most common lower limb abnormalities in children [1]. It is defined as a medial deviation of the leg’s mechanical axis relative to the thigh, producing an outward curvature of the leg even when the ankles touch [2]. This condition is normal in babies due to their position in the womb, but if a child still has bowlegs at the age of three, they should be evaluated by an orthopedic specialist to determine if further action is necessary [3,4]. Infants commonly exhibit physiological bowing of the lower extremities until approximately two years of age. This condition is typically symmetrical and painless, often accompanied by internal foot rotation and an increased tendency to trip. Fortunately, it generally resolves spontaneously as the child grows and develops [5,6]. Parental education and periodic clinical follow-up are usually sufficient to monitor the natural resolution of the deformity. In the meantime, simple interventions such as reversing the shoes may help reduce the frequency of tripping [7].

Although the exact prevalence of genu varum is unknown, it is sufficiently familiar to be considered a normal physiological variation in toddlers. However, it is also a significant reason why parents seek medical attention from their primary care providers [7,8]. It is essential that primary care providers possess adequate knowledge of lower limb deformities to appropriately assess and triage these cases. Orthopedic consultation is only necessary in the most persistent or concerning cases. While not mandatory, X-rays may be required to distinguish physiologic varus from pathologic conditions that require treatment. Genu varum is a condition with multiple causes and is broadly classified into physiological and pathological categories [2,9]. Parents often worry about this condition, which can be caused by a variety of factors [10]. The severity of the deformity can vary greatly, ranging from relatively harmless physiological bowing that can be reassured and observed to more complex cases [11].

Physiological genu varum is usually self-limiting and may only require clinical follow-up [12]. However, in pathological situations, the condition often worsens over time, increasing the risk of joint complications and mechanical issues in the lower limbs. The most common type of pathologic bowlegs is Blount's disease, also known as tibia vara, which must be distinguished from physiologic genu varum [13]. Treatment for Blount's disease typically requires orthopedic intervention to address the increasing varus deformity. Early detection of the condition can spare patients from invasive procedures, improve their prognosis, and reduce the potential consequences [14]. Genu varum is one of the most common lower limb musculoskeletal variations in Saudi Arabia, accounting for 24% of anomalies, alongside genu valgus [15]. Despite its common occurrence, parents and primary care physicians could lack awareness and knowledge regarding the distinction between physiological and pathological genu varum [16]. This gap in understanding can lead to unnecessary anxiety, delayed medical consultations, or inappropriate management of the condition. Therefore, in Saudi Arabia, where cultural and social factors may influence health-seeking behaviors, it is crucial to assess parents' knowledge and awareness of genu varum. This understanding of parents’ knowledge and perception can help identify areas where educational interventions are needed to improve early recognition and appropriate management of the condition. Improving parental awareness could reduce unnecessary anxiety, promote timely medical consultations, and ensure that children with pathological genu varum receive appropriate care. Thus, this study aimed to fill this gap by evaluating the level of knowledge and awareness among Saudi parents regarding physiological genu varum. It also assesses the parents’ discrimination between physiological and pathological genu varum.

## MATERIAL AND METHODS

### Study design and setting

This study employed a questionnaire-based cross-sectional design to assess knowledge and perceptions related to genu varum among Saudi parents. The study was conducted in Saudi Arabia, targeting parents across all regions of the country. Data collection took place electronically from May to December 2023, utilizing Google Forms. The questionnaire was disseminated through various social media platforms to reach a wide audience of eligible participants.

### Identification of the study population

Participants were enrolled in the study through a convenience sampling method. The study population consisted of parents aged 18 years or older living in Saudi Arabia who were married, widowed, or divorced. Participants were identified through the demographics section of the questionnaire.

### Study site

The study was conducted online, with participants recruited from all regions of Saudi Arabia. The electronic nature of the study enabled nationwide participation, resulting in a diverse and representative sample.

### Inclusion and exclusion criteria

The inclusion criteria were parents aged 18 years or older, living in Saudi Arabia at the time of the study, and individuals who were married, widowed, or divorced. The exclusion criteria were single individuals or those living outside Saudi Arabia at the time of the study.

### Recruitment of study participants

Participants were invited to participate in the study through social media platforms, including Twitter (X), WhatsApp, and Facebook. The questionnaire link was shared in parenting groups, forums, and other relevant online communities. A clear explanation of the study's purpose and eligibility criteria was provided to potential participants to ensure informed consent.

### Minimization of participant bias

To minimize bias, the study ensured anonymity by not collecting personally identifiable information. Additionally, the recruitment strategy targeted diverse social media platforms to avoid overrepresenting specific demographic groups. We also targeted various parenting groups, including parents of children of different ages from all regions of Saudi Arabia.

### Sample size calculation

According to the Statistical Yearbook 2016, published by the General Authority for Statistics, the number of marriages recorded in Saudi Arabia in 2016 was 5,650,747. Using a 95% confidence interval, a 5% margin of error, and a design effect of 2, the minimum required sample size was calculated to be 385 participants.

### Instruments and data collection

Data for this study was collected electronically using a structured questionnaire developed in Arabic and divided into three sections: 1) demographics (age, gender, education level, and marital status), 2) knowledge of genu varum (participants' understanding of genu varum and its physiological aspects), 3) perceptions of physiological genu varum (participants' perceptions and attitudes toward the condition).

The questionnaire was developed based on the available literature and then underwent a rigorous validation process. A panel of three experts evaluated the questionnaire for content validity, ensuring that it aligned with the study's objectives. A pilot study involving 15 participants was conducted to assess the reliability, clarity, and feasibility of the tool. Feedback from the pilot study was used to refine the questionnaire before its final deployment. The questionnaire trigger items demonstrated a satisfactory level of reliability, with a Cronbach's Alpha coefficient for the scale data of 0.76. Removing any of the questionnaire items did not improve the questionnaire’s reliability. So, all items were kept. The questionnaire was distributed via Google Forms.

## DATA ANALYSIS

The collected data were analyzed using the Statistical Package for the Social Sciences (SPSS, version 21; IBM Corp., Armonk, NY, USA). All statistical tests were two-tailed, with a significance threshold set at α = 0.05. A *P* value ≤ 0.05 was considered statistically significant. For each correct response related to knowledge and awareness, one point was assigned. Bowleg awareness was measured overall by adding together the discrete values for each correct awareness item. The total awareness score was calculated by summing the individual scores across all relevant items. An overall awareness level was considered poor if the participant's score was less than 60% of the total, and good if the score was 60% or higher. Descriptive statistics were used to present the frequency distributions and percentages for participant demographics, education level, and region of residence. Responses related to knowledge and awareness of physiological genu varum were tabulated, and overall knowledge levels were illustrated graphically. Cross-tabulations were performed to assess the association between participants’ knowledge levels and their demographic characteristics, using the Pearson chi-square test. When expected cell counts were low, the exact probability test was applied.

## RESULTS

A total of 1,222 participants were included in this study, with the majority being women (70.2%) and men (29.8%). The age distribution indicates an unbalanced spread among young participants, as those between 36 and 50 years old comprised the largest proportion of participants (42.3%), and those between 26 and 35 years old accounted for approximately one-third of the total respondents. The younger than 25 years old category accounts for a smaller fraction (9.3%).

In terms of education, a significant proportion of participants held a university degree (70.8%), followed by pre-university education (16%), and postgraduate degrees (10.3%) (Table 1). Regarding income, the data revealed a diverse distribution: participants earning between 3,000 and 10,000 SAR and 10,000 and 20,000 SAR monthly accounted for approximately two-thirds of the sample, whereas those with an income below 3,000 SAR represented the smallest group (7.2%). Geographically, the Western Region had the highest representation (42.0%), while the other regions contributed proportionally, with the Northern Region having the lowest participation (6.1%). In terms of nationality, the majority of participants were Saudi (92.7%), with a smaller fraction being non-Saudi (7.3%). The marital status indicated that most participants were married (91.2%), followed by those who were divorced (6.8%) and widowed (2%).

**Table 1:** Demographic characteristics of participants

Characteristics	Frequency	Percent (%)
**Gender**		
Male	364	29.8
Female	858	70.2
**Age**		
Younger than 25	114	9.3
26–35	401	32.8
36–50	517	42.3
> 50	190	15.5
**Educational level**		
No education	35	2.9
Pre-university	196	16.0
University	865	70.8
Postgraduate	126	10.3
**Region**		
Western Region	516	42.2
Southern Region	241	19.7
Eastern Region	256	20.9
Central Region	134	11.0
Northern Region	75	6.1
**Income**		
<3,000 SAR monthly	88	7.2
3,000–10,000 SAR monthly	455	37.2
10,000–20,000 SAR monthly	480	39.3
>20,000 SAR monthly	199	16.3
**Nationality**		
Saudi	1133	92.7
Non-Saudi	89	7.3
**Marital status**		
Married	1115	91.2
Divorced	83	6.8
Widow	24	2.0

Table 2 presents data related to children and parental knowledge of genu varum. The data provide insight into parental status, the presence of genu varum among their children, and parents' knowledge levels regarding the condition. Among the surveyed parents, 88.8% reported having children, while 11.2% did not. Of those with children, 15% stated that at least one child had genu varum, whereas the majority (85%) reported that their children did not have the condition. Regarding knowledge levels, 52.2% of parents demonstrated a poor understanding of the condition, while 47.8% showed a good understanding. This distribution indicates a need for improved parental awareness and understanding of genu varum, as a substantial proportion of respondents demonstrated poor knowledge about the condition.

**Table 2:** Distribution of factors related to children and parents’ knowledge about genu varum

Characteristics	Frequency	Percent (%)
**Do you have children?**		
Yes	**1,085**	**88.8**
No	**137**	**11.2**
**If yes, do any of your children have genu varum? (n = 1,085)**		
Yes	**173**	**15.0**
No	**980**	**85.0**
**Knowledge level about genu varum**		
Poor knowledge (below the average knowledge)	**540**	**52.2**
Good knowledge (above the average knowledge)	**494**	**47.8**

**Figure 1:**
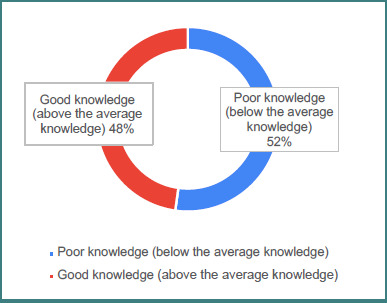
Level of parental knowledge about genu varum

**Figure 2:**
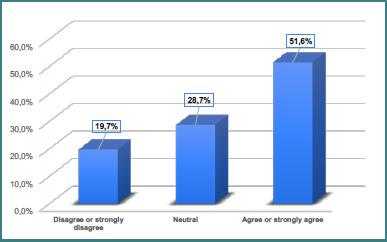
Genu varum (bowlegs) can be present normally in children up to 2 years

Table 3 presents the distribution of items related to parents' knowledge about genu varum (bowlegs) in children. The data reveal several trends in parental awareness (Figure 1). A majority of parents correctly recognize that genu varum may occur normally in children (54.3% agree or strongly agree) and that it is common in those under 2 years of age (63.6% agree or strongly agree). However, fewer parents (51.6%) agreed that genu varum is considered normal up to age two, indicating some uncertainty regarding developmental timelines (Figure 2). Parents were generally aware that physiological genu varum is mostly painless (56.1% agreed or strongly agreed), symmetrical (64.6% agreed or strongly agreed), and unlikely to cause walking difficulties (68.8% agreed or strongly agreed). Opinions were divided on the necessity of treatment: while 28.7% believed no treatment is required for physiological cases, 49.9% disagreed or strongly disagreed. Similarly, the majority recognized that factors such as genetics, bone structure, or fetal position in the womb can contribute to physiological genu varum (72.5% agreed or strongly agreed). Regarding the necessity of treatment, a large majority of parents understood that in severe cases, treatment such as braces or surgery may be necessary for physiological bowlegs (74.2% agreed or strongly agreed).

**Table 3:** Distribution of items related to parents’ knowledge about genu varum

Items	Frequency	Percent (%)
**Genu varum (bowlegs) can be present normally in children**		
Disagree or strongly disagree	254	23.6
Neutral	238	22.1
Agree or strongly agree	584	54.3
**Genu varum is common among children below 2 years of age**		
Disagree or strongly disagree	132	12.3
Neutral	260	24.2
Agree or strongly agree	684	63.6
**Genu varum (bowlegs) can be present normally in children up to 2 years of age**		
Disagree or strongly disagree	212	19.7
Neutral	309	28.7
Agree or strongly agree	555	51.6
**Physiological genu varum is mostly painless**		
Disagree or strongly disagree	118	11.0
Neutral	353	32.9
Agree or strongly agree	602	56.1
**Physiological genu varum is mostly symmetrical**		
Disagree or strongly disagree	111	10.4
Neutral	266	24.9
Agree or strongly agree	690	64.7
**Physiological genu varum causes walking difficulty**		
Disagree or strongly disagree	114	10.7
Neutral	219	20.5
Agree or strongly agree	734	68.8
**Physiological genu varum does not require treatment**		
Disagree or strongly disagree	536	50.1
Neutral	226	21.1
Agree or strongly agree	307	28.7
**Physiological genu varum can be caused by factors such as genetics, bone structure, or fetal position in the womb**		
Disagree or strongly disagree	75	7.0
Neutral	221	20.5
Agree or strongly agree	781	72.5
**In severe cases, treatment such as braces or surgery may be necessary for physiological bowlegs**		
Disagree or strongly disagree	80	7.5
Neutral	197	18.4
Agree or strongly agree	796	74.2

Table 4 outlines the findings of a log-linear model that aimed to predict parents' knowledge about genu varum based on various predictors. The results offer insights into how factors such as gender, age, income level, region, nationality, marital status, and whether the parents' children have genu varum influence their knowledge about the condition.

**Table 4:** Findings of the log-linear model for predictors of parents’ knowledge about genu varum

Predictors	Categories	Reference group	*P* value	Risk ratio	Lower limit (95% CI)	Upper limit (95% CI)
**Gender**	Male	Female	0.476	0.991	0.97	1.02
**Age**	Higher age-group	lower age group	0.137	0.989	0.98	1.00
**Income**	Higher income level	Lower income level	0.151	1.012	1.00	1.03
**Region**	Western Region	Northern region	0.049*	0.955	0.91	1.00
	Southern Region	Northern region	0.341	0.976	0.93	1.03
	Eastern Region	Northern region	0.256	0.972	0.93	1.02
	Central Region	Northern region	0.332	0.974	0.92	1.03
**Nationality**	Saudi	Non-Saudi	0.434	1.021	0.97	1.08
**Educational level**	Illiterate	Postgraduate	0.932	0.996	0.91	1.09
	Pre-University	Postgraduate	0.741	1.008	0.96	1.06
	University	Postgraduate	0.838	1.004	0.97	1.04
**Marital status**	Married	Widow	0.018*	0.896	0.82	0.98
	Divorced	Widow	0.062	0.91	0.82	1.01
**Do any of your children have genu varum?**	Yes	No	<0.001*	1.058	1.03	1.09

The analysis revealed that gender did not significantly impact parents' knowledge about genu varum, as indicated by a *P* value of 0.445. Similarly, age did not have a significant influence on knowledge, with a *P* value of 0.154. Income level also did not show a significant influence (*P* value > 0.005), suggesting that parents' knowledge about the condition was not related to their income.

Regarding regions of residence, parents from the Western Region tended to have significantly lower knowledge levels compared to those from the Northern region (*P* value = 0.049). In contrast, knowledge levels of parents from the Southern, Eastern, and Central regions did not differ significantly from those in the Northern region. Nationality did not significantly influence knowledge about genu varum, as indicated by *P* values of 0.365 and 0.066, respectively. However, marital status did show some significance, with less knowledge among married parents than among widowed parents.

One of the most significant findings concerns the association between parental knowledge and the likelihood of having a child diagnosed with genu varum. Parents whose children had the condition were more likely to have better knowledge about genu varum (*P* value < 0.001), with a risk ratio of 1.06. This indicates that direct exposure to the condition positively influences parental understanding.

In summary, the log-linear model in Table 4 demonstrates that factors such as region, marital status, and whether parents' children have children with genu varum play a more influential role in shaping parents' knowledge about the condition compared to factors like gender, age, income level, and nationality.

## DISCUSSION

Bowlegs, medically known as genu varum, is a type of deformity that is typically reported in children [17]. Although it is typically harmless and a normal phase of the physiological development of the lower limbs, it can be associated with underlying medical conditions [18]. It is crucial to carefully assess the patient and monitor the progression to ensure there is no underlying pathology.

Literature showed that the tibiofemoral angle in children follows a typical pattern as they grow, regardless of gender [19]. Firstly, a visible genu varum alignment is present before the age of 1 year, but at around 1.5 years old, the lower limbs begin to straighten. Afterward, a significant genu varum alignment develops in the child between the ages of two and three, which gradually resolves. By the time the child is 6–7 years old, they typically have a physiological genu varum of 5-6°, which remains as the skeleton continues to mature. However, Salenius and Vankaa also explained that there is a standard deviation of ±8° at any age [6]. Any deviation beyond these typical tibiofemoral angles could indicate an underlying pathological cause for the deformity and should be thoroughly investigated.

The current study aimed to assess parents' knowledge and awareness of physiological genu varum in Saudi Arabia. The study results revealed that nearly half of the parents were knowledgeable about physiological genu varum. In more detail, the majority of parents recognized that genu varum can be a normal finding in children and acknowledged its common occurrence in those under the age of two. More than half of the parents agreed that physiological genu varum is mostly painless, symmetrical, and unlikely to cause walking difficulty. However, uncertainty remained regarding the normality of genu varum up to the age of two and the necessity of treatment. Approximately one-quarter of parents believed that physiological genu varum does not require intervention, indicating a gap in understanding about when medical attention might be warranted. Regarding risk factors, parents demonstrated satisfactory awareness, with many recognizing that genetics, bone structure, and fetal position in the womb can contribute to the development of physiological genu varum. Nearly three-quarters of participants were aware that treatment options such as braces or surgery may be required in more severe cases. The study also revealed that married parents and those with a child with genu varum had significantly higher levels of knowledge compared to other groups.

To date, no prior studies have specifically assessed parents' knowledge or awareness of bowlegs (genu varum); however, several have explored parental understanding of related conditions, such as rickets. Alahmadi *et al*. [20] found that 27.9% of participants were knowledgeable about rickets, with 34.9% expressing perceived susceptibility, 54.5% acknowledging perceived severity, 25.9% recognizing perceived benefits, 11.7% identifying perceived barriers, and 5.4% responding to cues to action. Another study conducted in the Eastern Region of Saudi Arabia in 2021 revealed that nearly 62.8% of a total of 422 participants had insufficient knowledge regarding rickets, a condition that leads to skeletal abnormalities such as genu varum [21]. Similarly, in Egypt, Bishay *et al*. [22] reported that the low level of family awareness about genu varum was a significant factor contributing to the high prevalence of genu varum among children. Understanding genu varum and its risk factors within families is crucial for distinguishing between physiological genu varum and pathological genu varum, as well as for preventing the progression of the physiological type to pathological genu varum.

### Limitations

The study has several limitations that should be taken into consideration. First, the study relied on self-reported data, which had the potential for self-reporting bias, including recall bias. Parents may overestimate their knowledge or provide socially acceptable responses, which can potentially affect the accuracy of the results. Second, the study is prone to sampling bias. Although the study aimed for a diverse sample, the recruitment strategy through social media platforms may have excluded parents who did not use these platforms or have limited internet access. Third, the cross-sectional design limits the ability to establish causal relationships between variables. Fourth, the study was limited to Saudi parents, and generalizability to other populations may be limited. Additionally, the predominance of female participants may skew the results, as male perspectives may differ. Lastly, the study did not assess the long-term impact of parental knowledge on the management or outcomes of children with genu varum.

## CONCLUSION

In conclusion, the current study revealed that approximately half of the parents had satisfactory knowledge and information about genu varum, primarily regarding physiologic genu varum and its associated consequences. Higher knowledge was associated with being married and having a child with genu varum. Parental awareness about the disorder and its nature has not been previously discussed in the literature; therefore, larger-scale studies are needed that can cover all aspects of the disorder regionally and internationally. Additionally, health education campaigns for parents about that condition and how to manage it are also essential.
